# Structural characterization and adjuvant action of *Paulownia tomentosa* flower polysaccharide on the immune responses to classical swine fever vaccine in mice

**DOI:** 10.3389/fvets.2023.1271996

**Published:** 2023-09-19

**Authors:** Xiaolan Chen, Yaming Yu, Yi Zheng, Jiping Jia, Junjie Jin, Hongxiang Sun, Chunmao Jiang, Haifeng Yang

**Affiliations:** ^1^Jiangsu Agri-Animal Husbandry Vocational College, Taizhou, Jiangsu, China; ^2^College of Veterinary Medicine, Nanjing Agricultural University, Nanjing, China; ^3^Wenzhou Vocational College of Science and Technology, Wenzhou, Zhejiang, China; ^4^Zhejiang University, Hangzhou, Zhejiang, China

**Keywords:** *Paulownia tomentosa* flower polysaccharide, characterization, classical swine fever vaccine, adjuvant, Th1/Th2 immune responses

## Abstract

*Paulownia tomentosa* flower polysaccharide (PTFP) from dried cultured *P. tomentosa* flowers, is widely known for its immunomodulatory activities. Here, PTFP was extracted from *Paulownia tomentosa* flower using hot water extraction, followed by ethanol precipitation methods. Structural characterization of PTFP was revealed by scanning electron microscope, high-performance anion-exchange chromatography, gel chromatography, ultraviolet and infrared spectral. Meanwhile, adjuvant action of PTFT on the immune responses to classical swine fever vaccine in mice was evaluated to further proclaim the immune regulatory effect of PTFP. The results showed that PTFP was a type of heteropolysaccharide with a dense, rough surface and high molecular weight (667.02 kDa), mainly composed of glucose (30.93%), rhamnose (29.99%), galactose (15.66%), arabinose (6.95%), mannose (5.52%), and xylose (4.80%). The results of gel chromatography suggested that the molecular configuration of PTFP may be a spherical structure. The infrared spectrum results confirmed that the functional groups and chemical bond of PTFP contained -OH, O-H, C-H, C=O, C-O, etc. Moreover, PTFP exhibited obvious immune enhancement effect by improving concanavalin A (ConA), lipopolysaccharide (LPS), and CSFV E2-stimulated splenocyte growth and natural killer cell activity in CSFV-immunized mice. Similarly, the titers of CSFV E2-specific IgG, IgG1, IgG2a, and IgG2b antibodies and IFN-γ and IL-10 levels in CSFV-immunized mice were distinctly increased by PTFP treatment. Overall, PTFP was a macromolecular heteropolysaccharide primarily containing glucose and rhamnose, and possessed the auxiliary effect of immune enhancement on the immune responses to classical swine fever vaccine.

## 1. Introduction

Classical swine fever (CSF) is one of the most common reemergent, contagious, economically significant, multisystemic viral diseases in swine (1, 2), which is notifiable to the World Organization for Animal Health (OIE) due to its severe impact on the economy and public health (3). CSF virus (CSFV) is the only etiological agent of CSF (4, 5), and its genome has one open reading frame encoding a polyprotein that is cleaved into four structural and eight nonstructural proteins (6, 7). Since unique pathogenic structure, there are scarce treatment options for CSF, and vaccination attenuated live against CSFV is commonly the main means of preventing CSF infection (8). To enhance the efficacy of vaccines, adjuvant vaccines have obviously improved the effectiveness of immunization through inclusion in the immunization programs (9). Although adjuvants have been used in vaccines to enhance vaccine efficacy for almost a century, only a few adjuvants have been licensed (10). Therefore, the new, safe, and effective vaccine adjuvants are worthwhile further research and development.

Polysaccharides are a common type of biological macromolecule in traditional herbal medicine with various biological properties, including antiviral, antitumor, antioxidant, anti-inflammatory, hypoglycemic, and immunomodulatory properties that have received widespread attention (11–14). Polysaccharides usually can adjust innate and adaptive immunity and have been primarily applied in human health care, achieving significant results in improving immunological function (15–19). *Paulownia* (Scrophulariaceae) is a fast-growing deciduous tree with economic potential owing to its wood value and high biomass production (20). In the previous studies, a variety of biologically active components, such as polyphenols and polysaccharides, have been discovered in *Paulownia tomentosa*. *Paulownia tomentosa* flower polysaccharide (PTFP) is among the principal active components of *P. tomentosa*. Recently, PTFP has been proven to enhance the immune response of chicken Newcastle disease vaccine by promoting lymphocyte proliferation, increasing specific antibody titers and IFN-γ secretion, suggesting the potentiality of PTFP as an adjuvant to elevate vaccine potency (21). However, there have been no reports on the role of PTFP in other vaccine immunizations.

In the current study, we extracted PTFP from the flowers of *P. tomentosa* and revealed the structural characterization of PTFP for the first time. Subsequently, the extracted PTFP was orally administered to mice immunized with the CSFV vaccine to evaluate the adjuvant effect of PTFP on CSFV live attenuated vaccines and identify a potential candidate to be used as a novel immunologic adjuvant.

## 2. Materials and methods

### 2.1. Materials

Dried-cultured *P. tomentosa* flowers were collected from Bozhou Guoxin Pharmaceutical Co. Ltd. (Anhui, China). Live attenuated CSFV (rabbit source) was purchased from Pulike Biological Engineering INC (China). Sources of fetal bovine serum (FBS), RPMI-1640 medium, ConA, LPS, CSFV E2 protein (CSFV E2), antibodies (IgG, IgG1, IgG2a, and IgG2b), and cytokine ELISA kits (IFN-γ and IL-10) are listed in Supplementary Table S1.

All the chemicals used in this study were analytical grade. The human leukemia cell line K562, which was sensitive to NK cells, was obtained from the Shanghai Institute of Cell Biology, Chinese Academy of Sciences. The cells were cultivated in the logarithmic phase of growth in RPMI 1640 medium supplemented with penicillin (100 IU/mL), streptomycin (100 g/mL), and FBS (10%) at 37°C and 5% CO_2_.

### 2.2. Preparation of PTFP

PTFP was extracted using a previously described method (21). Briefly, *P. tomentosa* flowers were decocted and filtered twice for 2 h and 1 h in water. Subsequently, the suspension condensed from the merged decoction was precipitated four times for 12 h in 95% ethanol. Finally, the samples were centrifuged and concentrated to a specific volume with 48% carbohydrate (D-glucose as the standard).

### 2.3. Scanning electron microscopic (SEM) of PTFP

The morphology of PTFP was observed by SEM (Zeiss Merlin Compact, Germany). The PTFP, coated with a thin gold layer, were laid on the placode, and then the images were analyzed by Sanshu Biotech. Co., LTD (Shanghai, China) under high vacuum at a magnification of 1,000 ×, 2000 ×, 5,000 × and 10,000 × fold.

### 2.4. Determination of monosaccharide composition in PTFP

PTFP with 5 mg was weighed accurately and placed in a sealed tube. Trifluoroacetic acid solution was added to the sample for acid hydrolysis, after hydrolysis, nitrogen gas was used to dry the residual PTFP in the tube. Wash with methanol, then blow dry and repeat methanol washing 2–3 times. The residue was dissolved in deionized water and passed through 0.22 μm microporous membrane filtration. The PTFP filtrate was analyzed by high-performance anion exchange chromatography on a CarboPac PA-20 anion exchange column using a pulse current detector. Data were collected on ICS5000+ (Thermo Technology) and processed using chromeleon 7.2 CDS (Thermo Science).

### 2.5. Detection of PTFP molecular weight

PTFP was dissolved in NaNO_3_ aqueous solution (0.1 mol/L), and filtered through a 0.45 μm pore size filter. Furthermore, PTFP was also dissolved in dimethyl sulfoxide (DMSO) solution containing lithium bromide at a density of 1 mg/mL, then filtered through a 0.45 μm pore size filter. The homogeneity and molecular weight of various fractions in PTFP were determined by using SEC-MALLS-RI. The weight, polydispersity index and number-average molecular weight of PTFP various fractions in NaNO_3_ aqueous solution or DMSO solution were measured by DAWN HELEOS-II laser photometer. Meanwhile, three tandem columns were provided and held at 45°C by using a model column heater. A differential refractive index detector (Optilab T-rEX, Wyatt Technology Co., United States) was simultaneously connected to give the concentration of fractions and the refractive index increment (dn/dc) value. The dn/dc value of the fractions in NaNO_3_ aqueous solution was measured to be 0.141 mL/g, and in DMSO solution was determined to be 0.07 mL/g. Data were acquired and processed using ASTRA6.1 (Wyatt Technology).

### 2.6. Ultraviolet and infrared spectral measurement of PTFP

The ultraviolet–vis spectrum of PTFP water solution (5 mg/mL) was determined in a wavelength range of 200–1,000 nm by using a multifunctional microplate reader (Multiskan GO, Thermo Fisher Scientific, United States). Under the same conditions, pure water was used as blank control by Sanshu Biotech. Co., LTD.

Fourier Transform infrared (FT-IR) spectra of PTFP was measured by using a spectrometer (Nicolet iZ-10, Thermo Nicolet, United States). PTFP was mixed with KBr powder and then pressed into 1 mm pellets for FT-IR measurement in the range of 4,000 to 400 cm^−1^ by Sanshu Biotech. Co., LTD.

### 2.7. Experimental animals

Male and female ICR mice were obtained from Shanghai Slake Laboratory Animal Co., Ltd., China. The mice were 5 weeks old and weighing 18–22 g, then allowed to acclimatize to the environment for one week for the following experiments. The mice were maintained in groups of less than five mice per cage under stable controlled conditions (24 ± 1°C, 50 ± 10% humidity, 12/12 h light/dark cycle), and food and tap water were freely available. The study was approved by the Animal Welfare and Ethics Committee of Zhejiang University (No. 18227).

### 2.8. Immunization treatment

**Figure 1:**
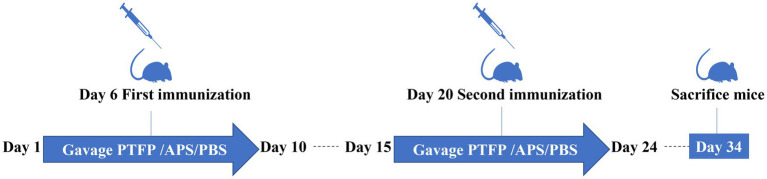
The schematic illustration of the vaccination and treatment schedule of the experiment.

ICR mice were raised in an environment free of specific pathogens, and separated into six groups and named: PBS, CSFV, APS, PTFP-L, PTFP-M, and PTFP-H groups, consisting of six male and six female mice each group. The experimental procedure was illustrated by schematic illustration (Figure 1). Except for the PBS group, the CSFV, APS, PTFP-L, PTFP-M, and PTFP-H groups were immunized twice by subcutaneous injection of 0.1 portion CSFV on day 6 and day 20 of the experiment. PBS and CSFV groups were gavaged with sterile PBS once a day. APS group was subjected to gavage APS (100 mg/kg) once a day. PTFP-L, PTFP-M, and PTFP-H were administered oral gavage at 30, 60, and 120 mg/kg doses of PTFP once a day, respectively. Throughout the experiment, PBS, APS, and PTFP were administered orally from day 1 to day 10 and from day 15 to day 24. On the day 34 of the experiment, mice were sacrificed by cervical dislocation, and the sera and splenocytes were reserved for further analysis.

### 2.9. Spleen proliferation assay

Splenocytes were separated as previously described (22). The spleen was aseptically removed and minced using scissors. The spleen tissue was then forced to pass through a fine steel mesh, and the cell suspension was collected. Erythrocytes were removed using ammonium chloride (0.8%, w/v). The collected splenocytes (5 × 10^5^ cells/well) were spread into 96 well plates in 100 μL complete medium and treated with classical swine fever virus E2 antibody (CSFV E2), concanavalin protein (ConA), or lipopolysaccharide (LPS) at 10, 5, and 10 μg/mL, respectively, while the blank medium was used as a control. After 44 h of culture at 37°C and 5% CO_2_, 50 μL MTT (2 mg/mL) was added to each well and cultured for a further 4 h. Subsequently, the cultured samples were centrifuged, and the supernatant was removed. A volume of 150 μL dimethyl sulfoxide was added to each well and incubated for 15 min. Finally, the absorbance was estimated at 570 nm using ELISA.

The stimulation index (SI) was measured according to the following equation:

$$\mathrm{SI}=A_{570}\;\left(\mathrm{CSFV E2,ConA or}\;\mathrm{LPS}\right)/A_{570}\;\left(\mathrm{control}\right).$$

### 2.10. Natural killer (NK) activity assay

The NK cell activity was evaluated by the MTT method (23). Specifically, K562 cells and splenocytes were defined as target and effector cells, respectively. K562 cells were seeded in 96-well U-bottom microtiter plates (2 × 10^4^ cells/well) in medium. Splenocytes were seeded at a density of 1 × 10^6^ cells/well, at an effector/target ratio of 50:1. After 20 h, NK cell viability was evaluated using the MTT method as follows: NK cell viability (%) = [A570_T_ – (A570_S_–A570_E_)] / A570_T_ × 100. A570_T_, A570_S_, and A570_E_ were the absorbance values of the target control, test sample, and effector control, respectively.

### 2.11. Antigen-specific antibody assay

CSFV E2-specific IgG, IgG1, IgG2a, and IgG2b antibodies in sera were measured using indirect ELISA (24). Briefly, 96 well ELISA plates were coated with 100 μL of CSFV E2 antigen (2.5 μg/mL in carbonate solution) per well at 4°C overnight. ELISA plates were blocked for 2 h at 37°C. Then, 100 μL of serially diluted serum samples was placed into the plates and incubated for 2 h at 37°C. The plates were incubated with IgG, IgG1, IgG2a, or IgG2b antibodies at 1:8000, 1:6000, 1:4000, and 1:4000 dilutions, respectively. After incubation for 2 h at 37°C, 100 μL of TMB was added to the plates and 50 μL of 2 mol/L H_2_SO_4_ was added 15 min after incubation to stop the reaction. Between each step, the ELISA plates were washed three times. Absorbance was measured at 492 nm.

### 2.12. Cytokine measurements

Splenocytes (5 × 10^6^ cells/well) were cultured with CSFV E2 (10 μg/mL) in 24-well plates. After incubation at 37°C in 5% CO_2_ for 72 h, the plate was centrifuged (2,500 rpm, 5 min), and supernatants were reserved for measurement of IL-10 and IFN-γ using ELISA kits (25).

### 2.13. Statistical analysis

All data were presented as mean ± standard deviation (mean ± SD). Statistical significance of differences was evaluated by ANOVA with Tukey’s post-hoc test. The significance threshold was set at *p* < 0.05.

## 3. Results

### 3.1. Morphological properties of PTFP

**Figure 2:**
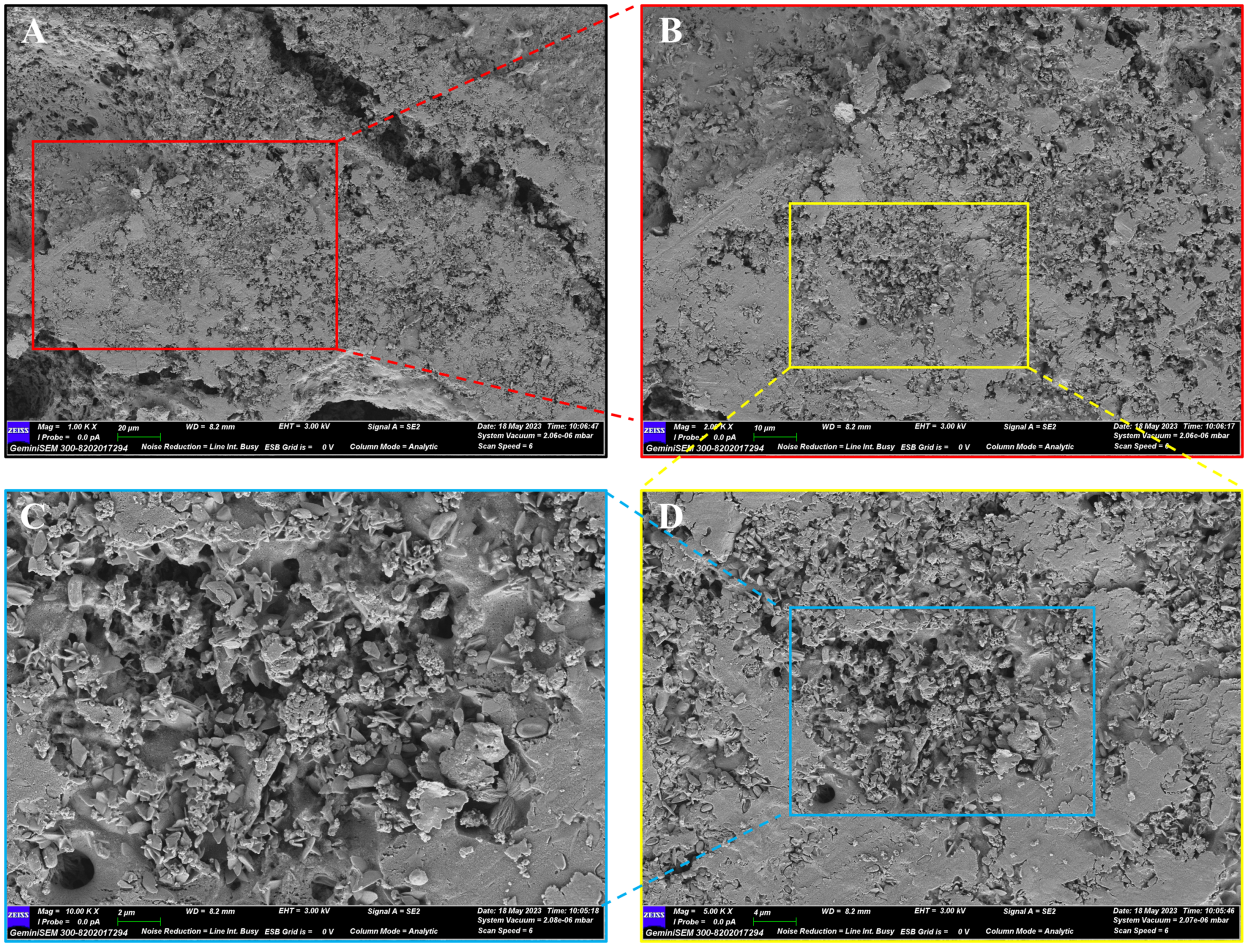
SEM diagram of PTFP. **(A)** × 1.00 k. **(B)** × 2.00 k. **(C)** × 5.00 k. **(D)** × 10.00 k.

The molecular morphology of PTFP was investigated by SEM under 1,000 ×, 2000 ×, 5,000 × and 10,000 × magnifications (Figure 2). The SEM results showed that the surface of PTFP was dense and rough, with a uniform dense layered structure, small fragments, no curling, and certain parts presented stacking phenomenon, suggesting that the intermolecular force of PTFP was strong and its molecular weight was large.

### 3.2. Monosaccharide composition and molecular weight analysis of PTFP

**Figure 3:**
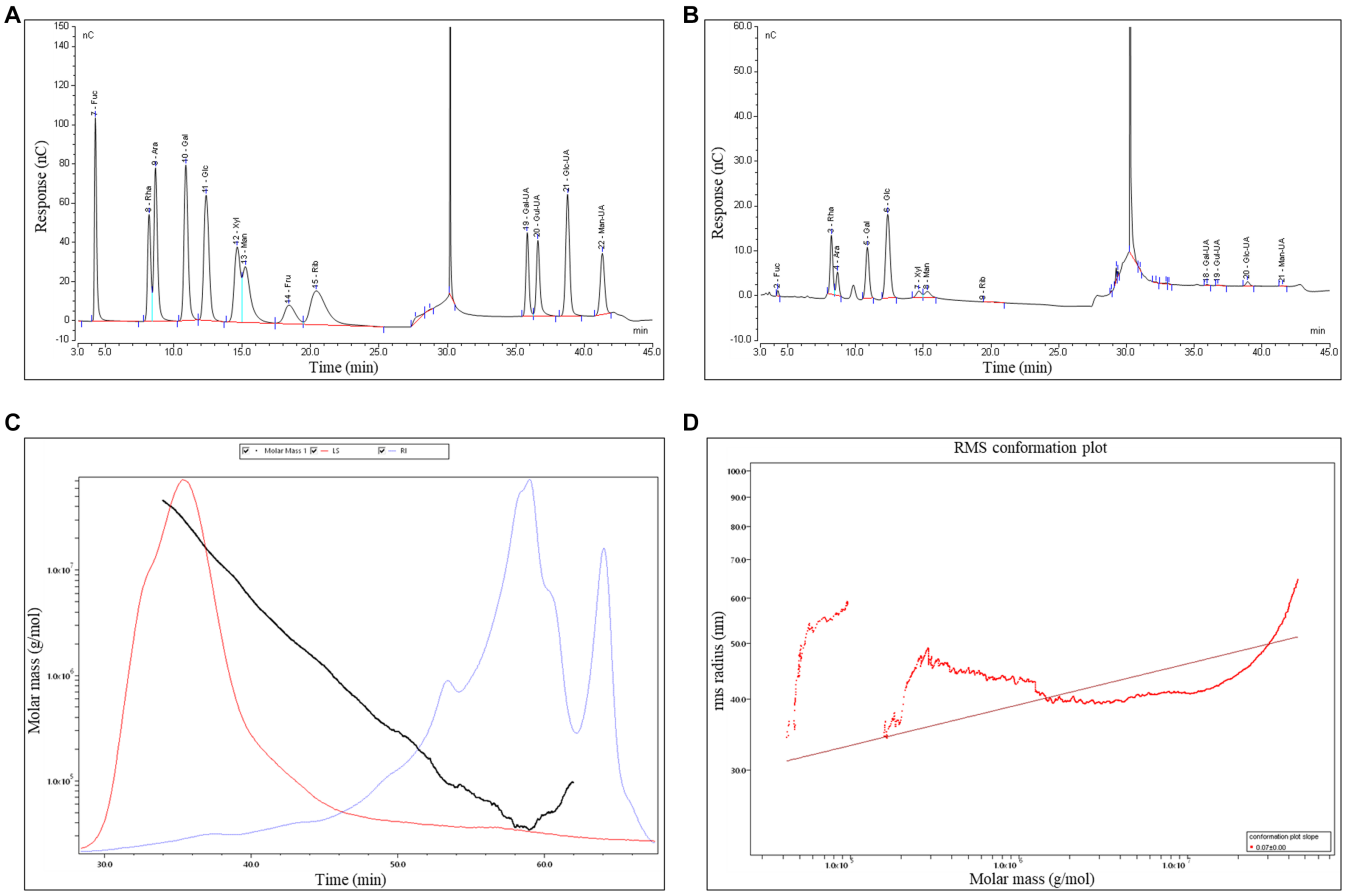
Analysis of the composition, molecular weight, and molecular configuration of monosaccharides in PTFP. **(A)** Standard ion chromatogram. **(B)** PTFP sample ion chromatogram. **(C)** Absolute molecular weight analysis of PTFP. **(D)** Molecular configuration analysis of PTFP.

The monosaccharides composition in PTFP was analyzed by HPAEC (Figures 3A,B). The specific quantification results are shown in Table 1. PTFP was primarily composed of glucose (Glc), rhamnose (Rha), galactose (Gal), arabinose (Ara), mannose (Man), and xylose (Xyl), and the molar mass ratio of their monosaccharide composition was 30.93, 29.99, 15.66, 6.95, 5.52, 4.80%. Based on the above results, it was indicated that PTFP was a heteropolysaccharide mainly composed of Glc and Rha.

**Table 1:** Analysis of monosaccharide compositions of PTFP.

Monosaccharide name	CAS number	Molecular formula	Monosaccharide composition (%)
Fucose (Fuc)	2,438-80-4	C_6_H_12_O_5_	1.09
Rhamnose (Rha)	10,030–85-0	C_6_H_14_O_6_	29.99
Arabinose (Ara)	5,328-37-0	C_5_H_10_O_5_	6.95
Galactose (Gal)	26,566–61-0	C_6_H_12_O_6_	15.66
Glucose (Glc)	50–99-7	C_6_H_12_O_6_	30.93
Xylose (Xyl)	58–86-6	C_5_H_10_O_5_	4.80
Mannose (Man)	3,458-28-4	C_6_H_14_O_6_	5.52
Fructose (Fru)	57–48-7	C_6_H_12_O_6_	0.00
Ribose (Rib)	50–69-1	C_5_H_10_O_5_	0.65
Galacturonic Acid (Gal-UA)	14,982–50-4	C_6_H_10_O_7_	0.71
Guluronic Acid (Gul-UA)	15,769–56-9	C_6_H_10_O_7_	0.69
Glucuronic Acid (Glc-UA)	6,556-12-3	C_6_H_10_O_7_	2.59
Mannuronic Acid (Man-UA)	6,814-36-4	C_6_H_10_O_7_	0.41

Gel chromatography was used to analyze the molecular weight of PTFP (Figures 3C,D). As shown in the Figure 3C, the molar masses ranged from 1.0 × 10^5^ to 1.0 × 10^7^ (g/mol) with symmetric signal peaks, suggesting that PTFP possessed a homogeneous relative molecular weight distribution. The specific molecular weights of PTFP are displayed in Table 2. Weight-average molecular weight (Mw) is the main indicator reflecting the mass of macromolecular substances. The Mw of PTFP was 667.02 kDa, indicating that PTFP possessed a large molecular weight. Polydispersity is the ratio of Mw to number-average molecular weight (Mn), represents the distribution range of molecular weight, and polydispersity of PTFP was 10.23, demonstrating the dispersion of molecular weight distribution of components in PTFP. Figure 3D displayed the molecular configuration of PTFP, and conformation plot slope was 0.07 ± 0.00, implying that the molecular configuration of PTFP may be a spherical structure. Moreover, the chain conformation of PTFP did not alter obviously with changes in molecular weight, which may indicate that PTFP had stable molecular conformation.

**Table 2:** Analysis of molecular weights of PTFP.

Distribution name	Value
Number-average molecular weight (Mn)	65.20 kDa
Peak-average molecular weight (Mp)	34.01 kDa
Weight-average molecular weight (Mw)	667.02 kDa
Z-average molecular weight (Mz)	16657.32 kDa
Polydispersity (Mw/Mn)	10.23

### 3.3. Ultraviolet and infrared spectral analysis of PTFP

**Figure 4:**
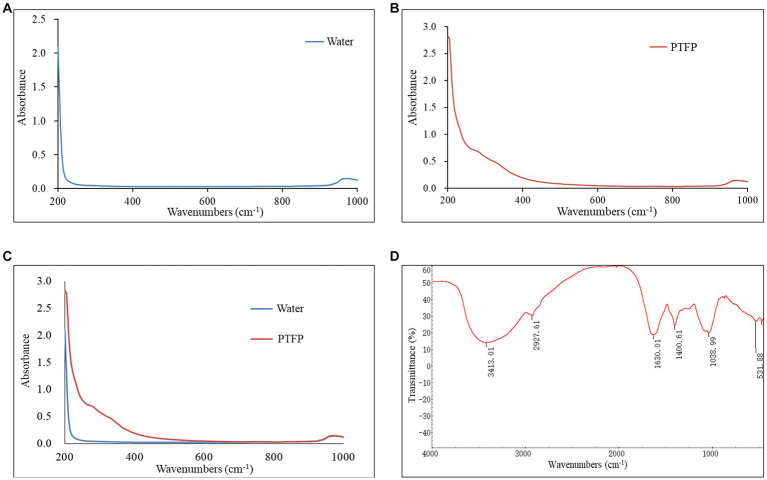
Analysis of ultraviolet and infrared spectrum of PTFP. **(A)** The curve of ultraviolet absorption spectrum of water as blank control. **(B)** The curve of ultraviolet absorption spectrum of PTFP. **(C)** The joint curve of ultraviolet absorption spectrum of water and PTFP. **(D)** Infrared spectral curve of PTFP.

The component and chemical bonds of PTFP were analyzed by ultraviolet and infrared spectrum (Figure 4). Compared with the blank control, the PTFP sample had absorption in the wavelength range of 200–400 nm, refreshing that the PTFP sample may contain small amounts of pigments, proteins, and nucleic acids (Figures 4A–C). As shown in the Figure 4D, the absorption band was the absorption peak of -OH stretching vibration at 3600–3200 cm^−1^, which was the characteristic absorption peak of sugars. The absorption peak at 3413.01 cm^−1^ was the stretching vibration O-H, representing the characteristic peak of sugars. The small absorption peak at 2927.61 cm^−1^ was attributed to C-H shearing vibration and bending vibrations of free sugars. The C=O stretching vibration at 1630.01 cm^−1^ suggested the existence of acetylation and uronic acid in PTFP. Furthermore, there was the absorption peak at 1038.99 cm^−1^, attributed to the stretching vibration of C-O.

### 3.4. Effect of PTFP on splenocyte proliferation

**Figure 5:**
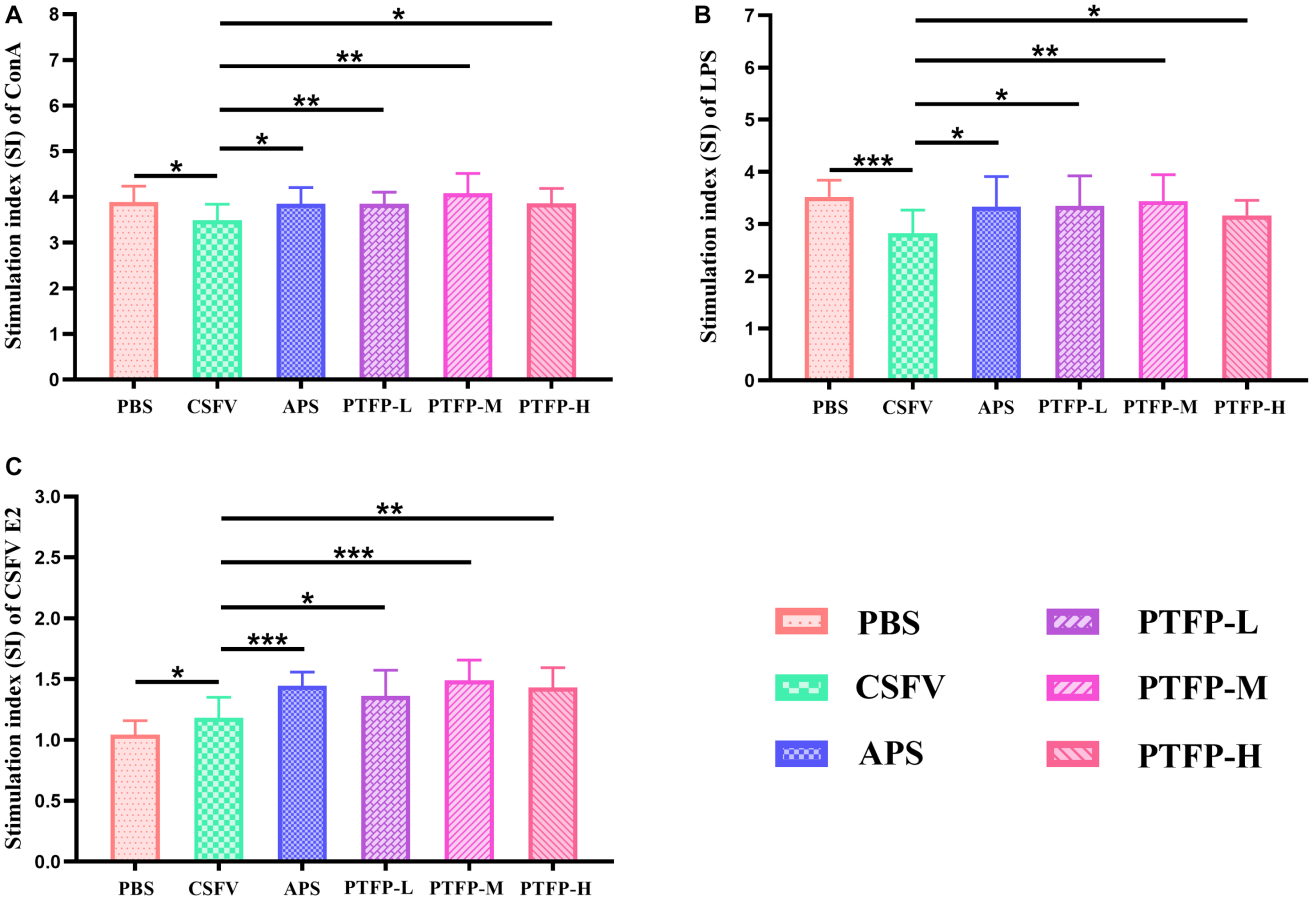
Effect of PTFP on splenocyte proliferation in the mice immunized with CSFV. **(A)** The SI was measured by stimulating splenocyte with ConA. **(B)** The SI was measured by stimulating Splenocyte with LPS. **(C)** The SI was measured by stimulating Splenocyte with CSFV E2.

The SI changes of splenocyte induced by ConA, LPS, and CSFV E2 are shown in Figure 5. SI is the main index reflecting the proliferation of splenocyte. Under ConA induction, compared with CSFV group, SI of splenocyte in PBS, APS and PTFP groups was significantly increased (Figure 5A) (*p* < 0.05 or *p* < 0.01), as was the case under LPS induction (Figure 5B) (*p* < 0.05, *p* < 0.01, or *p* < 0.001), suggesting that CSFV vaccine-immunized represented inhibitory effect on the proliferation of splenocyte after ConA or LPS stimulation, while APS and PTFP could promote splenocyte proliferation. Under CSFV stimulation, SI of splenocyte in APS and PTFP groups was significantly elevated compared to that in the CSFV group (Figure 5C) (*p* < 0.05, *p* < 0.01, or *p* < 0.001), whereas SI of splenocyte in PBS group showed a downward trend (*p* < 0.05), indicating that after stimulation with CSFV E2, proliferation of splenocytes from CSFV immunized mice could be promoted, with APS and PTFP showing remarkable effects. These data demonstrated that each dose of PTFP could significantly promote the proliferation of spleen cells induced by ConA, LPS, and CSFV E2 in mice immunized with CSFV, of which 60 mg/kg had the best effect that was equal to APS.

### 3.5. Effects of PTFP on NK cell activity

**Figure 6:**
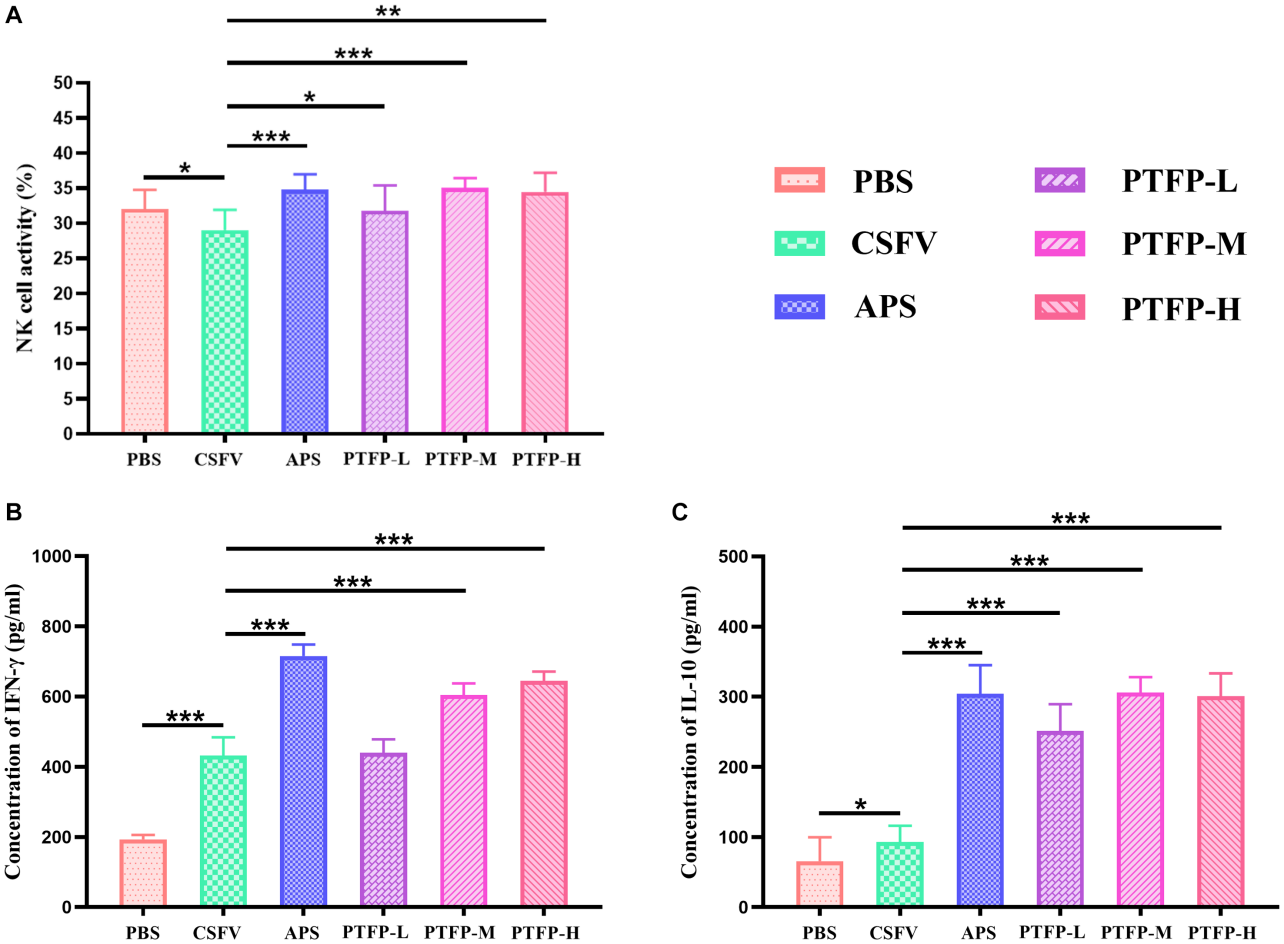
Effect of PTFP on NK cell activity and cytokine production in the splenocytes from the mice immunized with CSFV. **(A)** The NK cell activity in the splenocytes was measured by the MTT method. **(B)** Splenocytes were incubated with CSFV E2 protein, and the supernatants were collected for the detection of IFN-γ levels. **(C)** Splenocytes were incubated with CSFV E2 protein, and the supernatants were collected for the detection of IL-10 levels.

Figure 6A presents changes in NK cell viability, APS and PTFP at the three doses significantly promoted the activity of NK cells in CSFV-immunized mice (*p* < 0.05, *p* < 0.01, or *p* < 0.001). The findings revealed that all doses of PTFP could significantly activate NK cells in mice immunized with CSFV, and this effect was equivalent to that of APS (*p* > 0.05).

### 3.6. Effect of PTFP on cytokine secretion

Considering that cytokines play a pivotal role in the immune response and to explore the interaction between the Th1 cytokine IFN-γ and Th2 cytokine IL-10, we evaluated the effects of PTFP on the secretion of cytokines from CSFV E2-stimulated splenocytes. Compared with CSFV group, IFN-γ and IL-10 levels in PBS group were significantly elevated (*p* < 0.05, or *p* < 0.001). Moreover, APS and PTFP at doses of 60 and 120 mg/kg significantly increased IFN-γ levels (Figure 6B) (*p* < 0.001). APS and PFTP at three doses also significantly improved the ability of CSFV E2-stimulated splenocytes in CSFV-immunized mice to secrete IL-10 (Figure 6C) (*p* < 0.001).

### 3.7. Effect of PTFP on antigen-specific serum antibody responses

**Figure 7:**
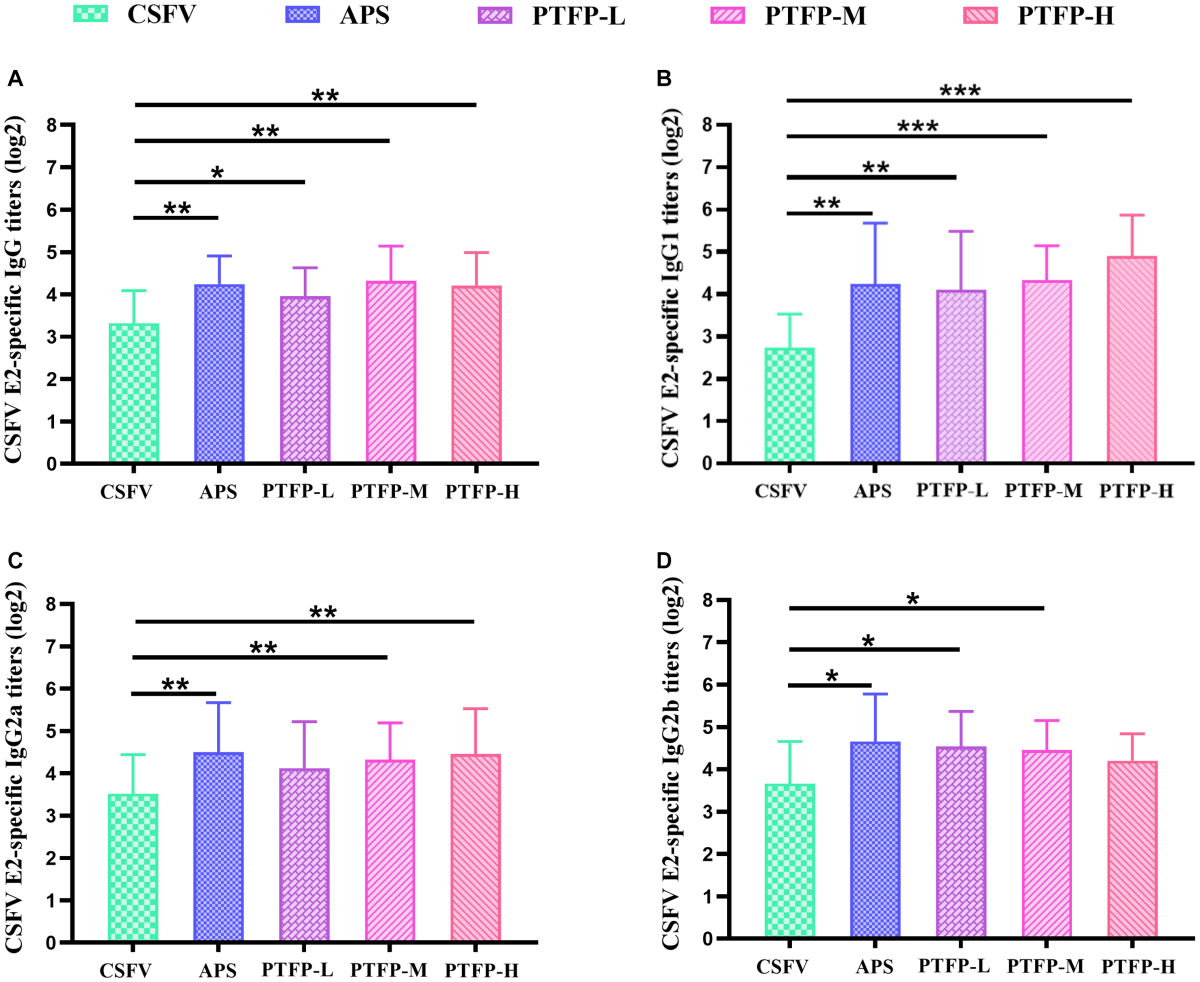
Effect of PTFP on CSFV E2-specific IgG and its isotype IgG1, IgG2a and IgG2b titers in the mice immunized with CSFV. **(A)** The titers of CSFV E2-specific IgG. **(B)** The titers of CSFV E2-specific IgG1. **(C)** The titers of CSFV E2-specific IgG2a. **(D)** The titers of CSFV E2-specific IgG2b.

As the nature of the antigen may play a pivotal role in the mediation of immune responses induced by an adjuvant, we explored the adjuvant effects of PTFP on antigen-specific humoral immune responses to CSFV E2. The CSFV E2-specific IgG, IgG1 antibody titers in CSFV-immunized mice were distinctly increased by APS and PTFP at each dose (Figures 7A,B) (*p* < 0.05, *p* < 0.01, or *p* < 0.001). Furthermore, APS and PTFP at 60 mg/kg significantly enhanced the IgG2a and IgG2b titers (Figures 7C,D) (*p* < 0.05). There was no obvious difference in the titers of CSFV E2-specific antibodies in sera between the three doses of PTFP, whereas the middle-dose group had the best effect equivalent to APS (*p* > 0.05).

## 4. Discussion

Polysaccharides isolated from various kinds of Chinese herbal medicines have gradually been proven to have lower side effects and various pharmacological actions, such as antioxidant, antiviral, antitumor and immunomodulator over the past few decades (17, 26). Therefore, the isolation, characterization, and pharmacological mechanism elucidation of polysaccharides from Chinese herbal medicines have become a hot research topic. By determining the characterization of polysaccharides, the structural information of active ingredients can be further clarified, which contribute to elucidate their pharmacological mechanisms at the molecular level. In the present study, PTFP was isolated from *P. tomentosa* flowers, and the SEM displayed that the surface of PTFP was dense and rough, indicating that PTFP possessed high molecular weight and strong intermolecular force. We further measured the molecular weight and configuration of the PTFP and confirmed that the Mw of PTFP was 667.02 kDa and the molecular configuration of PTFP was a spherical structure, which was consistent with the SEM results. Numerous studies reported that high molecular weight polysaccharides (> 100 kDa) were provided with better immune regulatory actions than low molecular weight polysaccharides (< 50 kDa), implying PTFP may have kind immune regulatory function (27, 28).

The composition of monosaccharide components is one of the fundamental parameters for characterizing polysaccharides, mainly hinging on its wide or narrow scope (29). Polydispersity reflects the distribution range of components in polysaccharides, with a value of approximately 1 indicating a narrow distribution range (30). In this study, polydispersity of PTFP was 10.23, suggesting that the molecular weight distribution of PTFP components was scattered. We further demonstrated that PTFP was a heteropolysaccharide, mainly composed of Glc, Rha, Gal, Ara, Man, and Xyl, especially Glc and Rha with the highest molar mass ratio. Among them, Glc, as a substrate for glycolysis, produced intermediate metabolites, such as citrate, itaconate and succinate during metabolism, which played critical roles in activating immune cells (31). Additionally, polysaccharides primarily rich in Glc, Rha, Gal, Ara, etc. have been demonstrated to possess potent immunomodulatory activities (32, 33). That is to say, PTFP could possess immunomodulatory potential.

In recent years, research has mainly focused on polysaccharides obtained from Chinese medicinal herbs due to their value as novel immune adjuvants against infections (34, 35). For example, Astragalus polysaccharides (APS) was evaluated for its potential as an effective adjuvant in vaccines against Porcine reproductive and respiratory syndrome virus (PRRSV) or CSFV, and demonstrated that APS had immunomodulatory effects on pig peripheral blood mononuclear cells exposed to PRRSV or CSFV (36, 37). PTFP has been proven to enhance the immune effectiveness of Newcastle disease vaccine in chickens (21). The current study aimed to explore the adjuvant effects of PTFP in mice immunized with the CSFV vaccine. In splenocyte proliferation experiments, CSFV significantly decreased the responses of splenic lymphocytes to mitogen stimulation in the vaccine alone group, which was in accordance with previous studies (38). Nevertheless, PTFP could induce the initiation of splenic lymphocytes in CSFV-immunized mice in this study, which was a committed step in triggering non-specific and antigen specific cell-mediated immune responses, and may enhance the immune function of mice.

NK cells are a group of cytotoxic lymphocytes that have non-specific killing abilities against virus-infected target cells and certain tumor cells. Although NK cells are activated after being attacked by viruses and bacterial pathogens, they are mainly considered essential for clearing viral infections (39). T cells, including helper T cell, cytotoxic T cells, and regulatory T cell, among which helper T cells can secrete a variety of cytokines, such as IFN-γ, IL-10, etc. to sensitize specific immune responses (40). In the present study, CSFV vaccine immunization significantly inhibited NK cell activity and promoted the secretion of IFN-γ and IL-10, while PTFP markedly enhanced NK cell activity and further facilitated IFN-γ and IL-10 secretion. These results indicated that vaccination with CSFV could predominantly activate specific immune responses, and PTFP exhibited potent effects in promoting both non-specific and specific immune responses after vaccination. IFN-γ and an accelerated IgG2a, IgG2b, and IgG3 are typical for the Th1 cell response, while IL-10 and an increased IgG1 and IgA are characteristic of the Th2-type response (41, 42). To clarify whether Th1/2 cell-induced the secretion of specific antibody participated in the PTFP adjuvant, we further investigated IgG and its isotype IgG1, IgG2a and IgG2b titers in the mice immunized with CSFV. The results showed that PTFP obviously increased the antibody titers of IgG, IgG1, IgG2a, and IgG2b, proving that PTFP enhanced Th1- and Th2-type responses to CSFV in mice.

## 5. Conclusion

In summary, PTFP was a macromolecular heteropolysaccharide primarily containing glucose and rhamnose. PTFP, as an adjuvant for CSFV, could provide effective protection against CSFV by stimulating both humoral and cellular immune responses. Through this study, we obtained further knowledge on structural characterization and the adjuvant value of PTFP in the prevention of CSF.

## Data availability statement

The original contributions presented in the study are included in the article/Supplementary material, further inquiries can be directed to the corresponding author.

## Ethics statement

The animal studies were approved by Animal Welfare and Ethics Committee of Zhejiang University (No. 18227). The studies were conducted in accordance with the local legislation and institutional requirements. Written informed consent was obtained from the owners for the participation of their animals in this study.

## Author contributions

XC: Writing – original draft, Writing – review & editing. YY: Writing – original draft. YZ: Data curation, Validation, Writing – review & editing. JiJ: Data curation, Validation, Writing – review & editing. JuJ: Writing – review & editing. HS: Methodology, Writing – review & editing. CJ: Conceptualization, Methodology, Writing – review & editing. HY: Project administration, Supervision, Writing – review & editing.

## Funding

The author(s) declare financial support was received for the research, authorship, and/or publication of this article. This study was financially supported by the National Natural Science Foundation of China (31702286), Tibet Autonomous Region Science and Technology Department Project (No. XZ202101YD0001C), Qinglan Project of Jiangsu Province (2021), Taizhou Science and Technology Supporting Agriculture Project (TN202006), Jiangsu Agri-animal Husbandry Vocational College Project (NSF2022ZR05).

## Conflict of interest

The authors declare that the research was conducted in the absence of any commercial or financial relationships that could be construed as a potential conflict of interest.

## Publisher’s note

All claims expressed in this article are solely those of the authors and do not necessarily represent those of their affiliated organizations, or those of the publisher, the editors and the reviewers. Any product that may be evaluated in this article, or claim that may be made by its manufacturer, is not guaranteed or endorsed by the publisher.
