# Supplementary material for: Structural characterization and adjuvant action of Paulownia tomentosa flower polysaccharide on the immune responses to classical swine fever vaccine in mice
Source: Front Vet Sci. 2023 Sep 19;10:1271996. doi: 10.3389/fvets.2023.1271996 (PMC10545964; doi:10.3389/fvets.2023.1271996)
Supplement: Supplementary file 1 [file Table_1.DOCX]

Supplementary Material

# Supplementary Tables

**TABLE S1** Source of materials used in the study.

| **Num.** | **Materials** | **Company/institutions** | **Region** |
| --- | --- | --- | --- |
| 1 | FBS | Gibco | USA |
| 2 | RPMI-1640 medium | HyClone | USA |
| 3 | ConA | Sigma-Aldrich | USA |
| 4 | LPS | Sigma-Aldrich | USA |
| 5 | CSFV-E2 | Shandong Binzhou Animal Science & Veterinary Medicine Academy | China |
| 6 | HRP-conjugated rabbit anti-mouse IgG antibody | Sigma-Aldrich | USA |
| 7 | HRP-conjugated goat anti-mouse IgG1, IgG2a, IgG2b antibodies | Southern Biotechnology Associates | SBA, USA |
| 8 | IFN-γ | BOSTER Biological Technology Co.,Ltd | China |
| 9 | IL-10 | BOSTER Biological Technology Co.,Ltd | China |

Notes: FBS, fetal bovine serum; ConA, Concanavalin A; LPS, lipopolysaccharide; CSFV, classical swine fever vaccine; HRP, horse radish peroxidase.
